# An Unusual Case of Acute Thrombosis of Abdominal Aortic Aneurysm without Acute Limb Ischemia

**DOI:** 10.1055/s-0038-1636991

**Published:** 2018-07-27

**Authors:** Spyridon N. Vasdekis, Sotiria Mastoraki, Andreas Lazaris, Konstantinos G. Moulakakis

**Affiliations:** 1 Department of Vascular Surgery, Attikon Hospital, School of Medicine, National and Kapodistrian University of Athens, Athens, Greece

**Keywords:** abdominal aortic aneurysm, thrombosis, ischemia

## Abstract

Acute thrombosis of an abdominal aortic aneurysm (AAA) is a rare and often devastating complication with high morbidity and mortality. In some cases, however, it may be associated with a silent course without signs of acute limb ischemia. The aim of this report is to describe an unusual case of acute thrombosis of AAA without signs of acute limb ischemia. Preoperative anxiety, stress, and phobia for surgery may be factors predisposing to acute thrombosis of an AAA.

## Introduction

Sudden thrombosis of an abdominal aortic aneurysm (AAA) is an uncommon condition. Its incidence is reported to be 0.6 to 2.8% of all surgically managed AAA cases. 1 2 3 Shumacker reported the first case of thrombosis of AAA in 1959, 4 and Jannetta and Roberts performed the first successful revascularization of thrombosed AAA in 1961. 5 For the most part, patients with thrombosed AAAs present with symptoms of acute limb ischemia, including pain, coolness, paresthesia, absent pulses, and mottling of the skin. 6

Several factors appear to be associated with acute AAA thrombosis: obstructive iliac disease with propagation of thrombus from occluded distal arteries, cardioaortic embolization, and accumulation of intrasaccular mural thrombus ultimately obstructing aortic flow. 2 7

The aim of this report is to describe an unusual case of acute thrombosis of AAA without signs of acute limb ischemia.

## Case Presentation

**Fig. 1:**
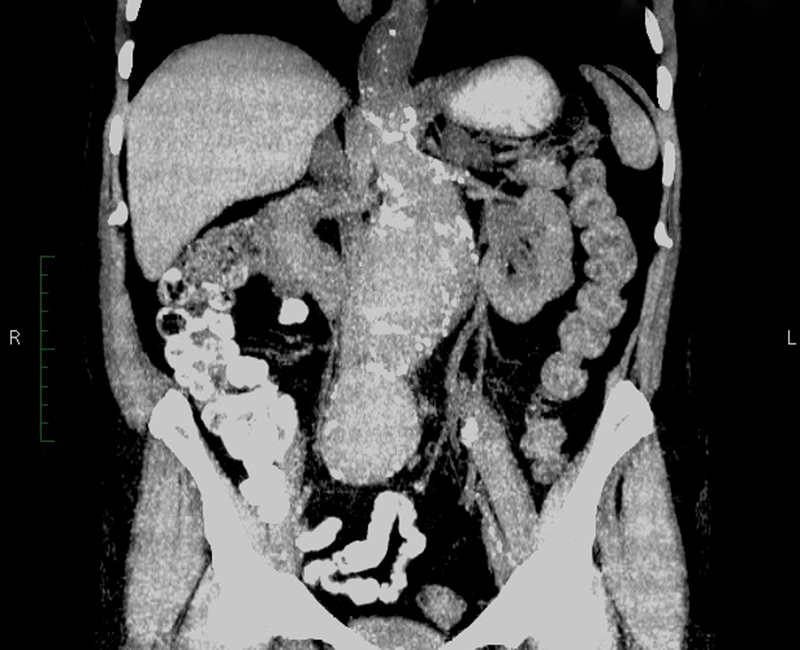
An asymptomatic juxtarenal 10 cm abdominal aortic aneurysm identified as incidental finding.

A 70-year-old woman presented in our vascular clinic due to an asymptomatic juxtarenal 10 cm AAA identified as an incidental finding in a recent computed tomography (CT) scan ( Fig. 1 ). Her medical history included mild hypertension under drug medication. At physical examination of the abdomen, a large pulsating mass was present with normal pulsation of femoral and tibial vessels. There were no complaints of previous intermittent claudication.

The woman was planned for open repair. She was very anxious about the result and the possible complications of the surgical procedure. The woman had intense stress, phobia for surgery, and was consulted by a psychiatrist.

**Fig. 2:**
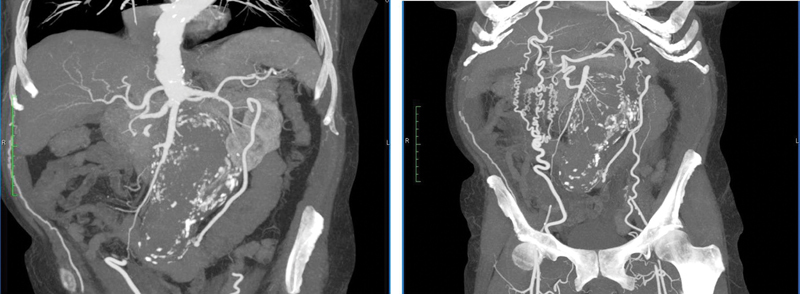
A computed tomography (CT) angiography 10 days after the first CT revealed an acute thrombosis of abdominal aortic aneurysm just below both the renal arteries with rich collateralization between the thoracic aorta and the common femoral arteries through the superficial epigastric arteries and other arteries of thoracic and abdominal wall.

To better define the aneurysm anatomy (with 1mm imaging slices), we performed a new CT angiography (CTA) 48 hours after admission, which surprisingly revealed complete thrombosis of the AAA just below both renal arteries without any signs of acute renal insufficiency, mesenteric ischemia, or limb ischemia ( Fig. 2 ). The most impressive element of the CTA was the rich collateralization between the thoracic aorta and the common femoral arteries through the superficial epigastric and other arteries of thoracic and abdominal wall. This collateralization was not evident in the first CT 2 days earlier.

The following physical examination revealed the absence of the previous pulsating mass and absence of femoral and distal leg pulses. Both legs were warm with normal skin color. The surgical procedure was postponed and the woman was discharged from the hospital with double antiplatelet therapy and weekly follow-up for the possible signs of limb ischemia.

After a month, the patient presented with severe intermittent claudication in the left lower limb. The following digital subtraction angiogram revealed a thrombosed abdominal aorta with collateral vessels between the aorta and both common femoral arteries ( Fig. 2 ). The woman underwent a left axillary–femoral bypass with polytetrafluoroethylene No. 8 graft. The patient's postoperative course was uneventful and the symptom of intermittent claudication disappeared. She was discharged on fourth postoperative day with antiplatelet (salicylic acid 100 mg, once daily) and statin (atorvastatin 20 mg, once daily) medication.

## Discussion

Acute thrombosis of AAA is a rare and often devastating complication of aortic aneurysms. Symptoms of acute lower limb ischemia (45.7%) associated with absent femoral pulses (68.6%) are the most common clinical signs. 2 3 Acute neurological deficits with lower limb paresis on both sides and paralysis have also been described. 8 9 In all reported cases, an emergent surgical intervention was performed to avoid an otherwise catastrophic outcome (death or limb loss). All patients were treated either with aortobiilliac/aortobifemoral bypass or with extra-anatomic revascularization of the lower extremities. Late rupture of thrombosed AAA has been reported in 15% of cases treated with axillobifemoral bypass. 10 11 12 13 Kumar reported successful endovascular treatment of a thrombosed aneurysm in a high-risk patient. 14

In our report, we present a case of acute thrombosis of AAA during hospitalization, which was not manifested with symptoms of acute limb ischemia. In a previously described “silent” presentation of an occluded AAA, a reconstruction with a tubular graft was performed. 15 In our case, impressive development of the rich collateralization through the superficial arteries of thoracic and abdominal wall in the time period of 10 days between the first CT and the CTA was noted. The lower limbs were viable without the need of an emergent surgical revascularization procedure.

There is no relationship between aneurysmal size and the likelihood of thrombosis. The transverse diameter of reported thrombosed AAA ranges from 3.5 to 10.5 cm. 6 According to the literature, several mechanisms that may explain an acute thrombosis of an AAA have been described:

1. Acute low-flow state due to occlusive iliac artery disease
2. A hypercoagulation disorder or hypercoagulability due to a neoplasm 3 16
3. A cardioaortic embolization due to cardiac arrhythmias causing an occlusion of the inflow or the outflow of the aneurysm
4. A dislocation of a fragment of the mural thrombus within the aneurysm sac causing an occlusion of the outflow of the aneurysm
5. A hypotension and low flow state due to hemorrhage, fever, dehydration, or other cardiac causes

Interestingly, in our case the patient was very anxious about the dangers of aortic reconstruction. We speculate that acute stress caused by the recognition of a large AAA and the possible complications of the planned surgical procedure may have predisposed to an acute thrombosis of the AAA. It is known that stress-related hormones (adrenocorticotropic hormone and cortisol) influence platelet-mediated thrombosis. 17 These hormones may fulfill an important role in acute arterial thrombosis by increasing the platelet aggregation. Moreover, according to a recent report, a poststress situation may increase D-dimers and clotting factors such as FVII:C, FVIII:C, FXII:C, and von Willebrand factor antigen. 18

In conclusion, acute thrombosis of AAA, although a complication with high morbidity and mortality, in some cases may be associated with a silent course without signs of acute limb ischemia. Preoperative anxiety, stress, and phobia for surgery may be factors predisposing to acute thrombosis of an AAA.
